# Cryo-soft X-ray tomography as a quantitative three-dimensional tool to model nanoparticle:cell interaction

**DOI:** 10.1186/s12951-016-0170-4

**Published:** 2016-03-03

**Authors:** Michele Chiappi, José Javier Conesa, Eva Pereiro, Carlos Oscar Sánchez Sorzano, María Josefa Rodríguez, Katja Henzler, Gerd Schneider, Francisco Javier Chichón, José L. Carrascosa

**Affiliations:** Centro Nacional de Biotecnología (CNB-CSIC), Cantoblanco, 28049 Spain Madrid,; National Heart and Lung Institute, Imperial College London, Exhibition Road, London, SW7 2AZ UK; MISTRAL Beamline-Experiments Division, ALBA Synchrotron Light Source, Cerdanyola del Vallès, 08290 Barcelona, Spain; Laboratory for Synchrotron Radiation–Catalysis and Sustainable Chemistry, Paul Scherrer Institut (PSI), 5232 Villigen, Switzerland; Institute for Soft Matter and Functional Materials, Microscopy Group Electron Storage Ring BESSY II, Helmholtz-Zentrum Berlin, Albert-Einstein-Str. 15, 12489 Berlin, Germany; Unidad Asociada CNB-Instituto Madrileño de Estudios Avanzados en Nanociencia (IMDEA Nanociencia), Cantoblanco, 28049 Madrid, Spain

**Keywords:** Cryo-soft X-ray tomography, SPION, Cancer cell lines, Hyperthermia, Endocytic pathways

## Abstract

**Background:**

Recent advances in nanoparticle design have generated new possibilities for nano-biotechnology and nano-medicine. Here we used cryo-soft X-ray tomography (cryo-SXT) to collect comprehensive three-dimensional (3D) data to characterise the interaction of superparamagnetic iron oxide nanoparticles (SPION) with a breast cancer cell line.

**Results:**

We incubated MCF-7 (a human breast cancer cell line) from 0 to 24 h with SPION (15 nm average diameter, coated with dimercaptosuccinic acid), a system that has been studied previously using various microscopy and bulk techniques. This system facilitates the validation and contextualization of the new 3D data acquired using the cryo-SXT-based approach. After vitrification, samples tested by correlative cryo-epifluorescent microscopy showed SPION accumulation in acidic vesicles related to the endocytic pathway. Microscopy grids bearing MCF-7 cells were then analysed by cryo-SXT to generate whole cell volume 3D maps. Cryo-SXT is an emerging technique that benefits from high X-ray penetration into the biological material to image close-to-native vitrified cells at nanometric resolution with no chemical fixation or staining agents. This unique possibility of obtaining 3D information from whole cells allows quantitative statistical analysis of SPION-containing vesicle (SCV) accumulation inside cells, including vesicle number and size, distances between vesicles, and their distance from the nucleus.

**Conclusions:**

Correlation between fluorescent microscopy, cryo-SXT and transmission electron microscopy allowed us to identify SCV and to generate 3D data for statistical analysis of SPION:cell interaction. This study supports continuous transfer of the internalized SPION from the plasma membrane to an accumulation area near the cell nucleus. Statistical analysis showed SCV increase in number and size concomitant with longer incubation times, and therefore an increase in their accumulated volume within the cell. This cumulative effect expands the accumulation area and cell organelles such as mitochondria are consequently displaced to the periphery. Our 3D cryo-SXT approach demonstrates that a comprehensive quantitative description of SPION:cell interaction is possible, which will serve as a basis for metal-based nanoparticle design and for selection of those best suited for hyperthermia treatment, drug delivery and image diagnosis in nanobiomedicine.

**Electronic supplementary material:**

The online version of this article (doi:10.1186/s12951-016-0170-4) contains supplementary material, which is available to authorized users.

## Background

Cancer is a multifactorial, heterogeneous disease and a major cause of death in developed countries [1, 2]. Classical therapeutic procedures, although well established, are based on radical practices and drugs that affect both normal and cancer cells. In recent years, superparamagnetic iron oxide nanoparticles (SPION) have emerged as one of the most promising tools to bypass the side effects of classic therapeutics, by taking advantage of their special magnetic properties. SPION can be used for bionanomedicine in many ways, as biosensors, for diagnosis [magnetic resonance imaging (MRI)] or for biomedical treatments such targeted cancer therapy guided by magnetic fields or combined with local release of anti-cancer drugs for theranostic approaches [3–5]. Superparamagnetic properties also allow SPION use in hyperthermia treatment, which produces an increase in local temperature when they are exposed to a variable magnetic field. Hyperthermia can be also combined with magnetic targeting and drug delivery [6].

Factors such as size, shape and surface charge determine nanoparticle behaviour as well as their fate inside the cell [6, 7]. The nanoparticle surface is therefore modified by adding biologically active compounds to facilitate their transport through the system to specific targeted cells, and to drive therapeutic agents that can be released locally. These features and their tuneable magnetic properties make SPION a particularly attractive tool for bionanomedicine. In addition to targeting drug delivery, which increases cure specificity [8], coated SPION are also retained by the cell for relatively long periods (72 h) with no signs of toxicity [3], a property crucial for hyperthermia treatment.

The use of cell cultures provides information essential for understanding cell:nanoparticle interactions prior to more complex in vivo analyses [9]. Qualitative microscopy studies reported SPION internalisation and accumulation through endocytic pathways [8, 10], as well as reduction in SPION superparamagnetic capabilities once internalised by the cell [11]. Achieving the full potential of functionalised SPION will nonetheless require precise quantitative analysis at sufficient resolution to model the interactions between nanoparticles and the cell environment.

Here we used near-native correlative microscopy techniques to characterise SPION behaviour in MCF-7, a human breast cancer cell line [10, 12], taking advantage of this well characterised SPION:cell model with no reported toxicity. To avoid cell drying, sectioning or staining artefacts, we used live confocal video-microscopy to study SPION dynamics at short incubation times (up to 3 h). To study SPION accumulation at longer incubation times, we used correlative cryo-epifluorescence microscopy and cryo-SXT [13, 14]. The technique is based on high X-ray penetration of biological samples and the natural contrast of cell structures in the water window energy range to obtain tomographic datasets at nanometric resolution (40–60 nm) [15–17]. In this study, we present quantitative information for intracellular SPION distribution and accumulation in MCF-7 cells, including accumulation rates and distances within the SCV. Our results complement previous information obtained using more qualitative approaches, and support the use of cryo-SXT as an essential tool to define nanoparticle:cell interaction. This information permits the design of in silico experiments such that the superparamagnetic properties of different nanoparticles can be tested in conditions similar to those characteristic of the cell environment.

## Results and discussion

### SPION uptake dynamics

**Fig. 1:**
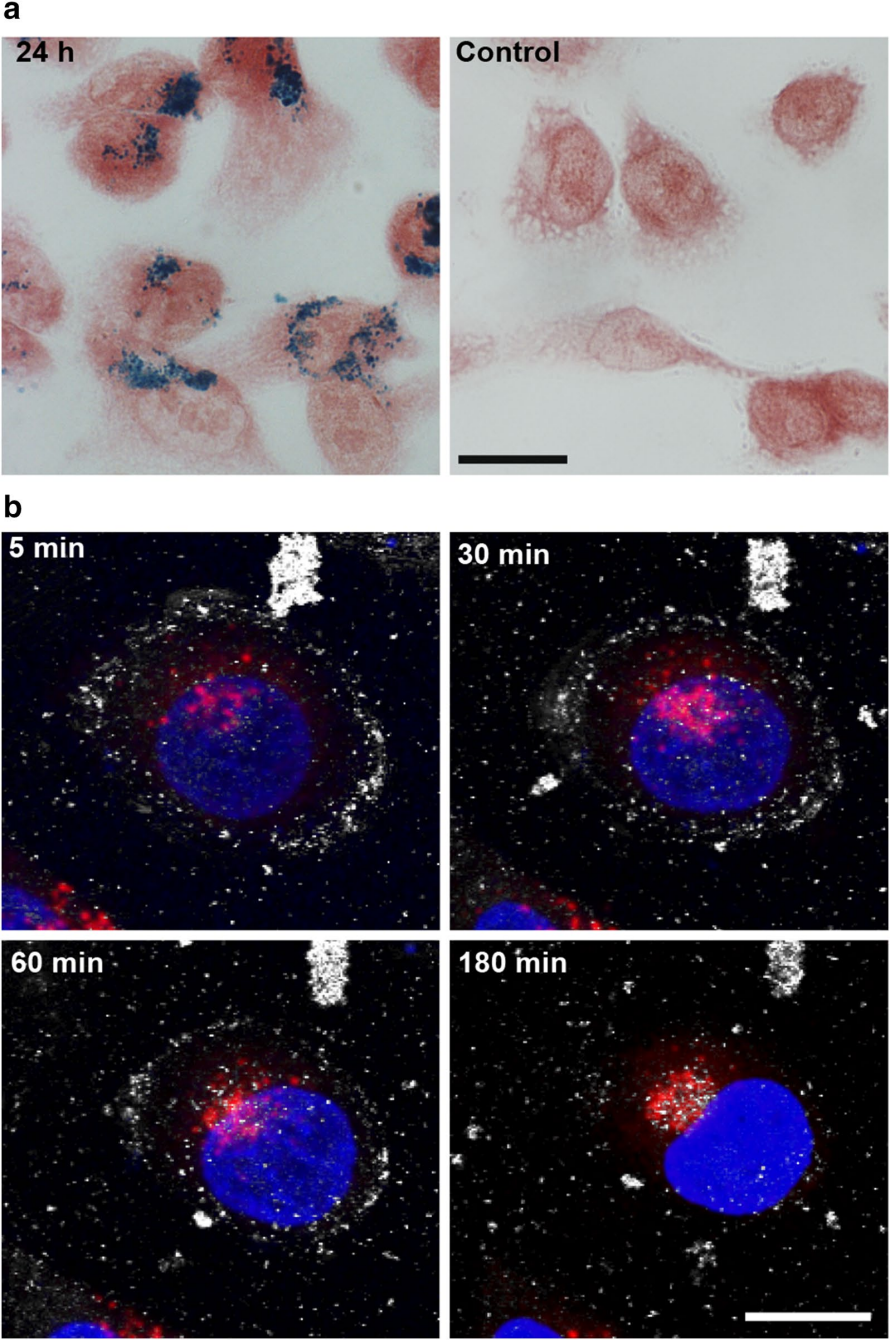
Cell uptake dynamics by optical microscopy. **a** Prussian blue staining and neutral red counterstaining of MCF-7 cells incubated for 24 h with 0.25 mg ml^−1^ SPION (*left*) and control cells without nanoparticles (*right*). *Bar* 20 μm. **b** Time-lapse confocal microscopy. Four confocal images of a SPION-incubated MCF-7 cell at 5, 30, 60 and 180 min. Nucleus, *blue* (DAPI), acidic vesicles, *red* (LysoTracker Red) and SPION, *white* (back-scattering light). *Bar* 10 μm

The MCF-7 breast cancer cell line was incubated with 0.25 mg ml^−1^ SPION for 24 h. A Prussian blue staining assay detected the nanoparticles in the vicinity of the cell nucleus as a blue precipitate, which was not observed in untreated control cells (Fig. 1a). Using confocal video-microscopy, we followed SPION internalisation dynamics by tracking reflected light dispersed by nanoparticle aggregates for 180 min (Fig. 1b; Additional file 1: Movie 1). After 5 min incubation with nanoparticles, light-reflecting clusters formed a halo around the cells. After 30 min, we detected an increase in the red fluorescence signal from LysoTracker Red-labelled acidic organelles. At 60 min, SPION clusters surrounded the nucleus, and at the end of the experiment (180 min), the cytoplasmic reflection signal had faded and reflection spots began to appear in the perinuclear area near the acidic network, which also increased. These results support the idea of rapid nanoparticle transport through the endocytic pathway to the accumulation area, a specific region near the nucleus related to endosomal degradation (Additional file 2: Figure S1A–C).

### Cryo-soft X-ray tomography

**Fig. 2:**
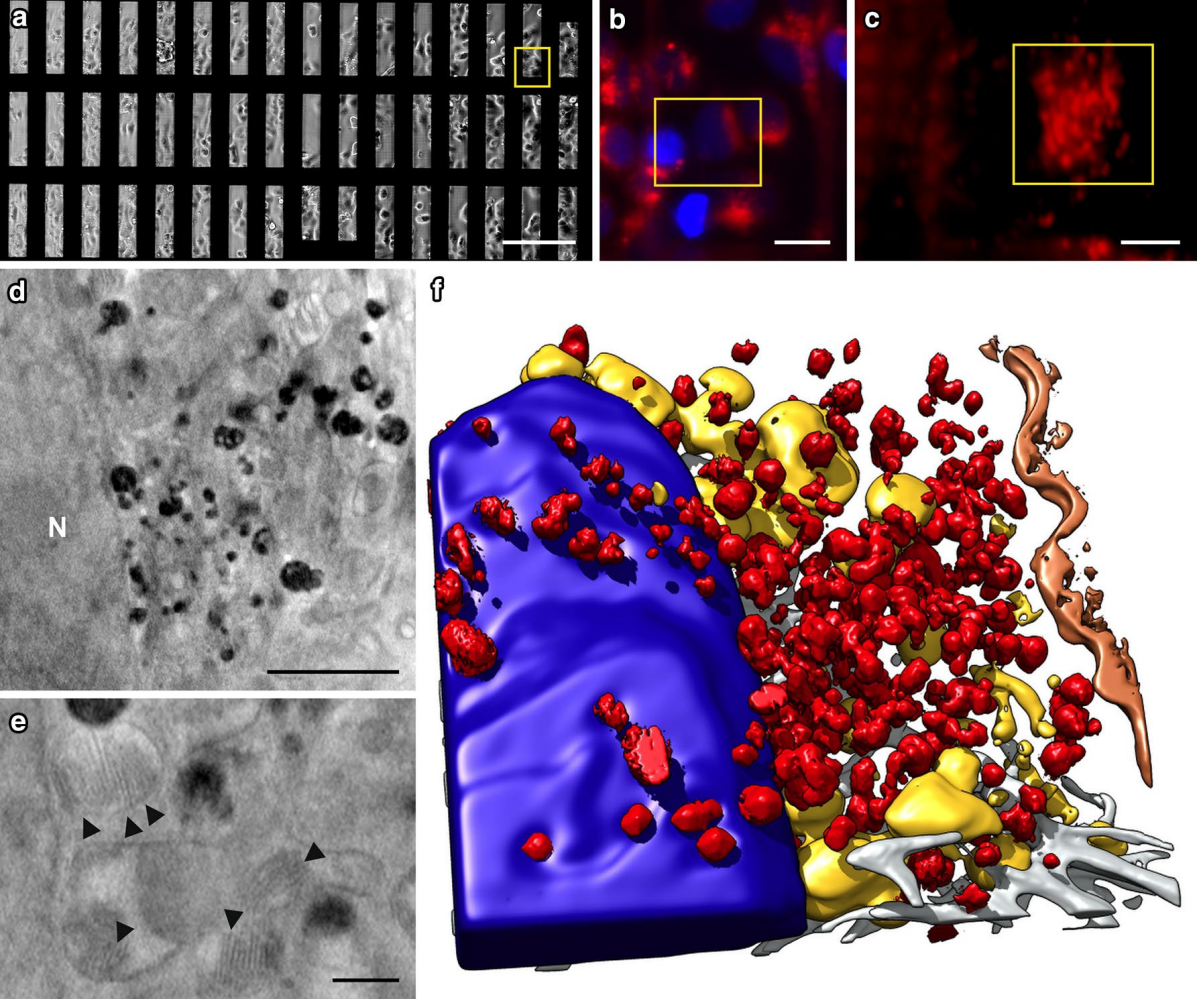
Fluorescent and cryo-SXT correlative workflow. **a** In vivo differential interference contrast (DIC) image of MCF-7 cells cultured on Au-HZBII grid and incubated 24 h with SPION (0.25 mg ml^−1^). *Bar* 200 μm. **b** In vivo fluorescent image from the area in the *yellow square* in **a**. *Bar* 20 μm. Nucleus, *blue* (DAPI), acidic vesicles, *red* (LysoTracker Red). **c** Cryo-epifluorescent image (*red channel*) from the area in the *yellow squar*e in **b**. *Bar* 5 μm. **d** Cryo-SXT plane from the area in the *yellow square* in **c**. N, nucleus. *Bar* 2 μm. **e** Cryo-SXT plane showing ultrastructural details of the cell. *Arrowheads* indicate mitochondrial cristae. *Bar* 500 nm. **f** Volumetric representation of the tomogram in **d**. High-absorption vesicles (*red*), segmented applying a threshold adapted to the volume containing the highest densities, are condensed near the nucleus (*blue*), displacing the mitochondrial network (*yellow*). *Grey* filaments, *orange* plasma membrane. Dataset acquired at HZB-BESSYII

MCF-7 cells were cultured on transmission electron microscopy (TEM) grids (Fig. 2a), labelled with fluorescent probes for correlative light/soft X-ray tomography (CLSXT), incubated with SPION for different times, and vitrified. Samples were imaged with the soft X-ray microscope in cryo-conditions (see “Methods” section).

We used correlative microscopy to acquire cryo-SXT tilt series of the specific LysoTracker-labelled areas in which SPION tend to accumulate, as shown by confocal experiments (Fig. 1; Additional file 2: Figure S1A–C). These areas were first imaged in live cells (Fig. 2a, b) and after cell vitrification, in cryo-conditions (Fig. 2c) to assure that no cell rearrangement was induced by vitrification (Additional file 3: Figure S2). Reconstructed cryo-SXT volumes had a resolution of ~60 nm, sufficient to visualise mitochondrial cristae (Fig. 2d, e, arrowheads). We also observed other cellular components such as intermediate filaments, actin bundles (Fig. 2f, grey) or plasma membrane (Fig. 2d, f, brown), as well as organelles such as the nucleus, including nucleolus and chromatin condensations (Fig. 2d, f; Additional file 4: Figure S3).

Cryo-soft X-ray tomograms of SPION-incubated MCF-7 cells showed an increase in high-absorption clusters at longer incubation times, which correlated with the LysoTracker Red signal (Fig. 2; Additional files 2 and 4: Figures S1D–F and S3). Three-dimensional reconstruction of whole cells showed high-absorption clusters concentrated mainly near the nucleus, although they were also found scattered throughout the cytoplasm; they were never found inside the nucleus (Fig. 2f; Additional file 4: Figure S3). These results coincide with the increase in SPION-loaded endocytic vesicles reported using classical 2D techniques [10, 12]. Volumetric representation of cells showed mitochondrial exclusion to the cell periphery caused by high-absorption cluster accumulation near the nucleus (Fig. 2f, yellow; Additional file 5: Movie 2). The high-absorption clusters inside cells had a non-homogeneous internal substructure, consistent with the segmented high-absorption voxel isosurface rugosity (Fig. 2 and Additional file 5: Movie 2).

**Fig. 3:**
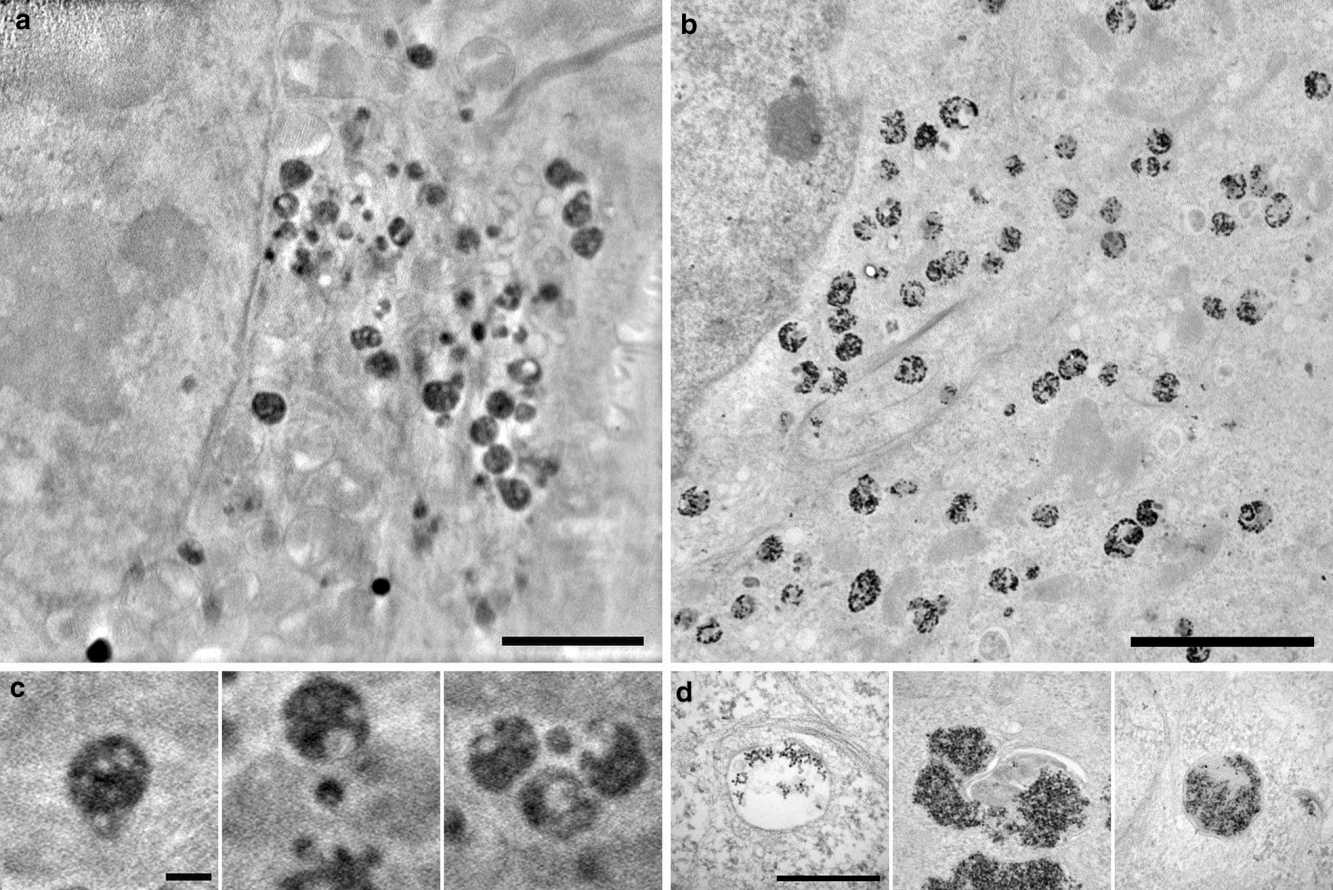
Ultrastructural comparison of cryo-SXT planes to TEM images. Cryo-SXT tomogram section (**a**) and TEM micrograph of a thin cellular section (**b**) of a MCF-7 cell treated with SPION for 24 h. **c** Cryo-SXT tomogram sections of high absorption vesicles extracted from a volume of a MCF-7 cell incubated with SPION for 24 h. **d** TEM micrographs of endosomal organelles containing SPION. *Bar* in **a** and **b**, 4 μm. *Bar* in **c** and **d**, 500 nm. Dataset acquired at HZB-BESSYII

As high-absorption clusters correlated with the LysoTracker Red signal, which also correlated with the scattered light signal from SPION (Additional file 2: Figure S1A–C), we compared cryo-SXT planes side-by-side with classical TEM micrographs in which SPION could be visualized directly. This comparison of cryo-SXT tomographic planes and TEM sections from cells incubated with SPION in equivalent conditions showed great similarities in morphological details despite of the differences in sample processing and image formation (Fig. 3a, b). Detailed comparison of cryo-SXT high-absorption cluster images (Fig. 3c) to images of the most common endocytic vesicles obtained by TEM (Fig. 3d) showed similarities in size, shape and internal substructure. These similarities and the fluorescent labelling of the acidic organelles, which correlates with high-absorption clusters in cryo-SXT and SPION-containing vesicles (SCVs) visualized by TEM (Additional file 2: Figure S1D–F and G–I, respectively), permitted the identification of high-absorption clusters in cryo-SXT as SCVs.

### Whole cell measurement and modelling

Retrieving whole cell ultrastructure in 3D by cryo-SXT produced data that enabled not only a qualitative description [18, 19], but also quantitative modelling of the cell-nanoparticle system. To model SPION:cell interaction, we collected SXT data from MCF-7 cells incubated for increasing periods of time. To analyse the enlargement of SCV with time, we measured their diameter manually (see “Methods” section). To parameterize the aggregation effect inside MCF-7 cells, we measured SCV distances to the nuclear membrane and calculated the distances between neighbouring SCV.

**Fig. 4:**
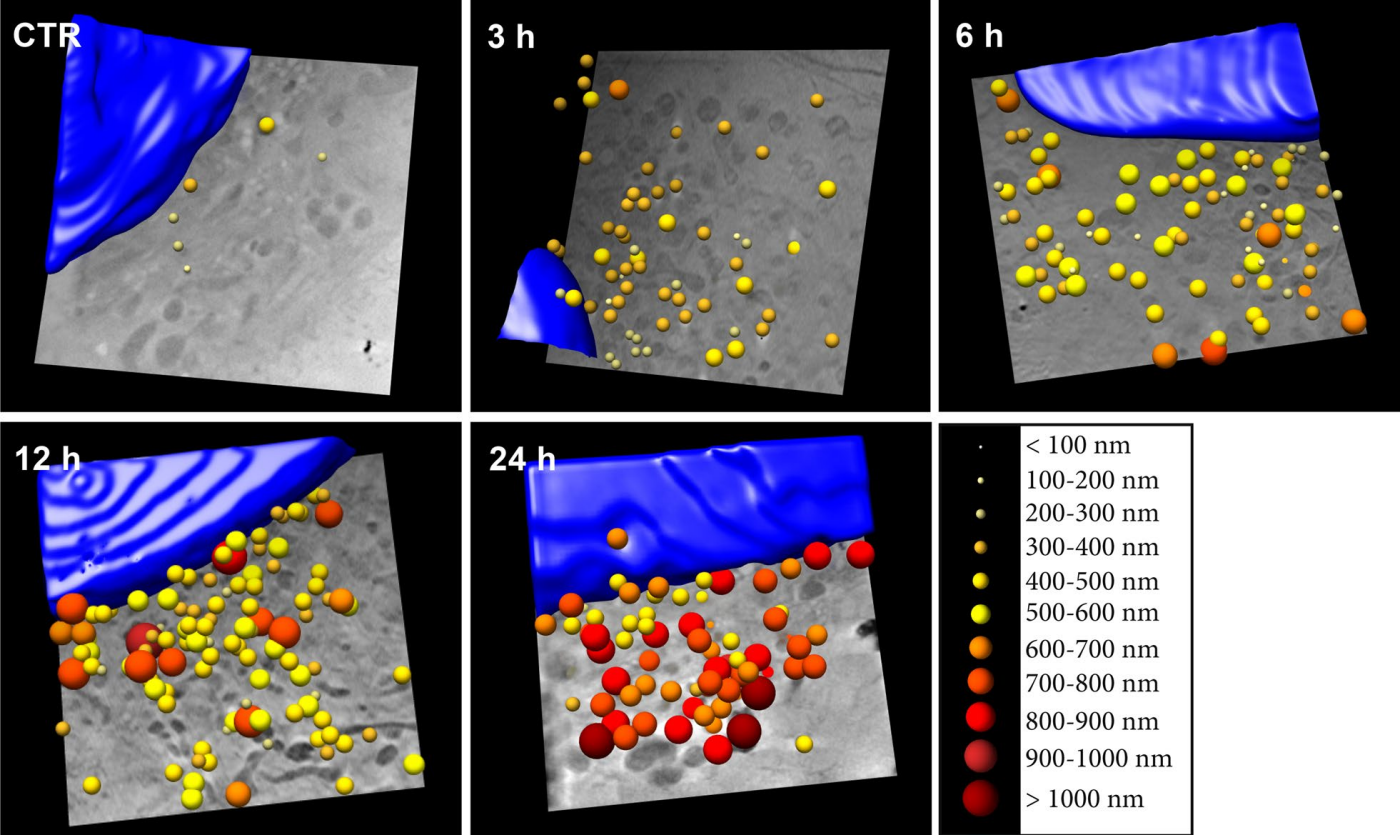
Cryo-SXT tomogram-derived colour-coded volumetric representation of endosome size in MCF-7 cells incubated with SPION for 0 (CTR), 3, 6, 12 and 24 h. The nucleus was segmented in *blue*. Field of view, 11.5 μm x 11.5 μm. Dataset acquired at ALBA

Using data extracted from the cryo-SXT tomograms of cells at different incubation times, we generated a color-coded size representation of SCV inside MCF-7 cells (Fig. 4). For each SCV coordinate, a sphere was drawn for the corresponding size range. Schematic vesicular 3D modelling showed qualitatively that vesicle number increased with incubation time, as well as clear SCV enlargement, with diameters from 600 nm (orange) to 1000 nm or more (dark red). We found a majority of large vesicles near the nucleus (dark red), but vesicles smaller than 600 nm (yellow) were also visible, even after 24 h incubation (Fig. 4).

### Quantification and statistics

**Fig. 5:**
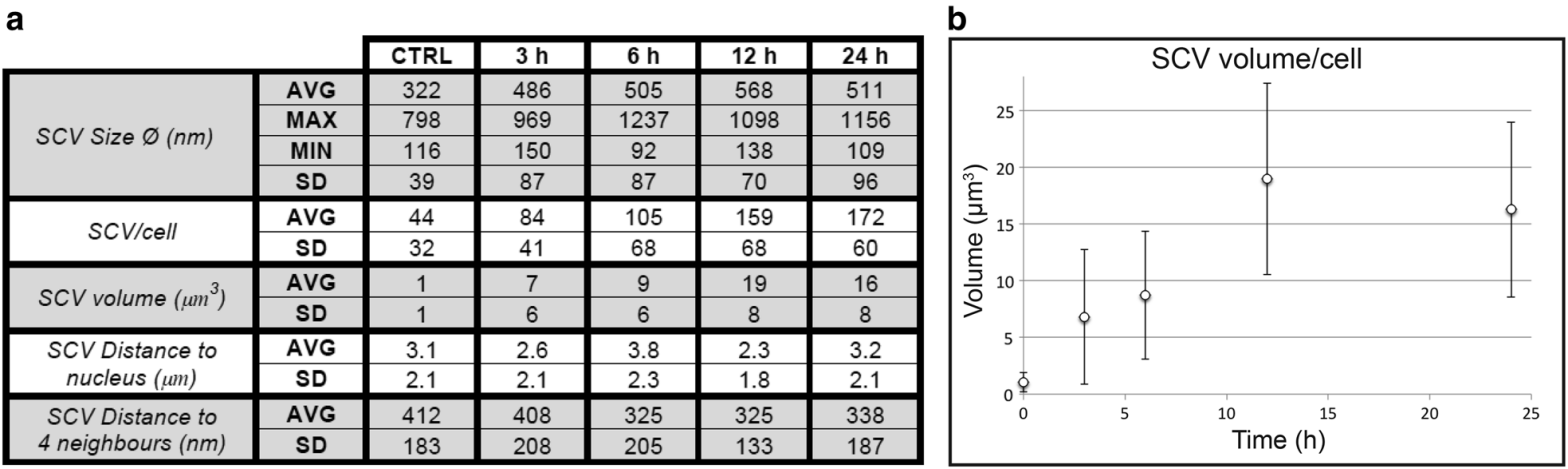
Statistics for SPION-incubated MCF-7 cells. **a** Statistical values for endosome distribution extracted from cryo-SXT at different cell incubation times. The table shows various parameters: mean (AVG), maximal value (MAX), minimal value (MIN) and standard deviation (SD). **b** Volume of SCV in MCF-7 cells during incubation

Cryo-SXT 3D data allowed statistical analysis of the SPION-incubated MCF-7 cells (Fig. 5a; Additional file 6: Figure S4). To facilitate analysis of size and volume, SCV were considered as spheres. Average SCV diameter increased from ~320 nm in control cells to ~570 nm after 12 h incubation (Fig. 5a; Additional file 6: Figure S4A). Maximum vesicle size at various incubation times nonetheless showed a constant value ~1 μm, an effect that might be related to possible stability of SCV. The minimal vesicle size of ~100 nm also remained stable throughout incubation, although this result is probably derived from the cryo-SXT resolution (near 60 nm using a 40 nm Fresnel zone plate lens), which makes unambiguous measurement of smaller vesicles difficult [20]. The standard deviation (SD) for size distribution was similar for measurements from 3 to 12 h post-incubation (87–70 nm) (Fig. 5a), but increased at 24 h (96 nm). In contrast, the SD for the control cell dataset was 39 nm, smaller than that computed for SPION-incubated cells. This effect is linked to MCF-7 cell cycle length, ~21 h [21]. SPION uptake is reported to slow down during cell division, and nanoparticle-loaded endosomes are distributed to daughter cells, thus decreasing endosome number (and total volume) per cell [22–24].

The analysis of mean SCV number per cell during incubation showed an increasing number of vesicles from ~85 at 3 h to ~170 at 24 h (Fig. 5a). The cell cycle appeared to affect this distribution less than for mean vesicle size, since the increase between 6 and 12 h was ~50 SCVs, and between 12 and 24 h, ~10 SCVs. The SD at 24 h was 60, slightly reduced in comparison with 70 at 12 h.

To quantify high-absorption cluster accumulation (combining SCV size and number), we calculated the total volume of SPION-loaded vesicles per cell (Fig. 5b). SCV intracellular accumulated volume showed a steady increase from 3 to 12 h (from ~7 to ~20 μm^3^), which indicated continuous SPION uptake, consistent with increased SCV number and enlargement. From 12 to 24 h, the intracellular accumulated volume remained almost constant. As discussed above, a possible explanation for this plateau is MCF-7 cell cycle length [22–24].

To study the aggregation effect over time, we analysed the distance from each SCV to the nucleus, which ranged from 2.3 to 3.8 μm (Fig. 5a; Additional file 6: Figure S4B). The invariability of this distance with incubation time suggested that SCV group stably in the accumulation area, whose position depended on the structure of each cell (Fig. 5a).

To analyse the behaviour of vesicles once they reached the accumulation area, we computed the distance of each SCV centre to its four nearest neighbours. The mean distance showed small variations, from ~412 nm in control cells to 325 nm in cells treated for 12 h (Fig. 5a; Additional file 6: Figure S4C). Distances remained almost constant for 3 h (~408 nm) and were reduced to 325 nm after longer incubation. At 24 h, we observed a slight increase in the distance, to 340 nm, again possibly due to a cell division effect. This hypothesis is supported by the SD increase at 24 h. SCV thus appeared to stabilise once gathered near the nucleus; the increase in the volume occupied by SCVs before cell division also supports continuous nanoparticle uptake throughout the cell cycle.

The accumulated volume increased during the incubation period. In contrast with the findings from qualitative microscopy studies (fluorescence optical microscopy and TEM [10]), SCV constant distance to the cell membrane and between SCVs suggest that vesicle maturation is not coupled to transport to the nuclear periphery, but rather takes place in the accumulation area.

## Conclusions

We present quantitative data at nanometric resolution of the fate of bioactive nanoparticles incorporated into cells. Beyond classical qualitative visualization, cryo-SXT tomograms and correlative microscopy of whole cells allowed us to compute size as well as the distance between SCVs at various incubation times.

Our results support a SPION uptake model in which the cell incorporates nanoparticles rapidly and continuously using endocytic vesicles, giving rise to an accumulation area near the nucleus. Once located in this area, SCV number and size increase. The total volume of the SCV per cell increased with SPION incubation time, ranging from ~7 to ~20 μm^3^ (at the maximum accumulation time, 12 h). This value represents ~1 % of the cell volume (~2000 μm^3^, assuming a 20 μm diameter and 3–5 μm thickness). We show that cryo-SXT is a valuable tool for generating a quantitative model to analyse the distribution and size of SCVs, which will lead to a better knowledge of the superparamagnetic properties of iron oxide nanoparticles. The SCVs have a mean size of ~570 nm at 12 h; they are separated from the nucleus by 2–3 μm and from each other by 0.3–0.4 μm.

The type of 3D information provided by cryo-SXT could be instrumental for generating in silico models for selecting the nanoparticles and incubation patterns best suited for hyperthermia applications, image diagnosis, and drug-targeted cancer therapies. The progressive increase in the use of engineered metal-based nanoparticles for cancer treatment will benefit from the implementation of cryo-soft X-ray tomography to track their fate and effects during their interaction with biological samples.

## Methods

### Magnetic nanoparticles

Dimercaptosuccinic acid-coated SPION of uniform size (15 nm) were obtained by thermal decomposition of an iron oleate complex in 1-octadecene, as described [12]. SPION were sterilised by 0.22 μm pore size filtration [Millex-GP, 0.22 μm, polyethersulphone (PES), syringe filter]. SPION stock at 4 mg ml^−1^ was dispersed by sonication (5 min) in a 40 kHz sonicator bath (Branson 3510 ultrasonic cleaner, Thomas Scientific). SPION were then resuspended in cell culture medium at a final concentration of 0.25 mg ml^−1^, the mixture was sonicated (1 min) and incubated with cells for different times.

### Cell culture

The human breast adenocarcinoma MCF-7 cell line was obtained from the American Type Culture Collection (HTB-22). Cells were cultured in Dulbecco’s Modified Eagle Medium (DMEM; Sigma–Aldrich) supplemented with 10 % (v/v) foetal bovine serum (FBS), 1 % penicillin/streptomycin (both from Gibco Life Technologies) at 37 °C in a humidified, 5 % CO_2_ atmosphere. MCF-7 cells were cultured on different supports depending on the experiment, including Petri dishes (Corning), 12 mm glass coverslips, glass-bottom Petri dishes (Ibidi) and holey carbon film-coated Au grids for EM (Quantifoil).

### Prussian blue staining and neutral red counterstaining

Cells were cultured on 12 mm glass coverslips; when they reached 80 % confluence they were incubated with nanoparticles. Cultures were fixed in methanol (−20 °C, 5 min), stained with an equal volume of 4 % hydrochloric acid and 4 % potassium ferrocyanide trihydrate (Panreac; 15 min), and counterstained with 0.5 % neutral red (Panreac; 2 min) for morphological analysis. After washing with milliQ water and air-drying, preparations were mounted in DePeX (Serva). Cells were visualised and imaged under a bright field microscope (Zeiss Axioscope).

### Time-lapse video-microscopy

For confocal microscopy, MCF-7 cells were cultured directly on 12 mm glass-bottom culture dishes (Thermo Scientific, Nunc). The fluorescent stain LysoTracker Red DND-99 (100 nM) was used to label acidic organelles, and DAPI nucleic acid stain (diluted 1:200; both from Molecular Probes, Life Technologies) to label the nucleus. Dyes were added to cell cultures ~30 min before video microscopy.

Samples were washed three times with PBS and the video-microscopy experiment began when 0.25 mg ml^−1^ SPION were added to cell medium. Fluorescence, light reflection (SPION back-scattering of light) and differential interference contrast (DIC) images of 4 confocal planes on a Leica TCS SP5 microscope (Leica Microsystems) with a 37 °C incubation system were taken every 2.5 min for the 3 h incubation with SPION. Videos were obtained by combining distinct sequential images (z axis projection merge of the 4 plane images) using LAS AF v.2.3.6 software (Leica Microsystems). For single image analyses, we used ImageJ software [25].

### Sample preparation for transmission electron microscopy

MCF-7 cells grown on coverslips in 24-well plates (BD Falcon) were incubated with 0.25 mg ml^−1^ SPION for different times. Samples were washed with PBS and fixed with a mixture of 2 % paraformaldehyde (Polysciences Inc.) and 2.5 % glutaraldehyde (TAAB Laboratories) in PBS (1 h, room temperature). The cell monolayer was washed with PBS and distilled water, post-fixed (45 min) with 1 % osmium tetroxide in PBS (TAAB Laboratories), washed with distilled water, treated (45 min) with 1 % aqueous uranyl acetate (Electron Microscopy Sciences), dehydrated with increasing concentrations of ethanol (SeccoSolv; Merck) and embedded in epoxy resin EML-812 (TAAB Laboratories; 2 day, room temperature). Resin-containing gelatin capsules (TAAB) were placed on coverslips and polymerised (2 days, 60 °C). Resin blocks were detached from coverslips by successive immersion in liquid nitrogen and hot water. Ultrathin 70 nm-thick sections were obtained with the Ultracut UCT ultramicrotome (Leica Microsystems), transferred to 200 mesh nickel EM grids (Gilder) and stained with 3 % aqueous uranyl acetate (20 min) and lead citrate (2 min). Sections were visualised on a JEOL JEM 1200 EXII electron microscope (operating at 100 kV). Micrographs were taken with a Gatan Erlangshen ES 1000 W digital camera at various magnifications.

### Epifluorescence microscopy

MCF-7 cells were cultured on Au-EM finder grids or Au-HZBII special grids for ALBA and HZB-BESSYII, respectively, coated with holey carbon (R 2/2; Quantifoil). When cells reached 70 % confluence, they were incubated with 0.25 mg ml^−1^ SPION for different times. LysoTracker Red DND-99 and DAPI were added 10 min before in vivo imaging of the grids. An automatic map of the grid was generated using a Leica DMI6000B epifluorescent microscope with an incubation system, using a 20×/0.4 Fluorotar L dry objective with an OrcaR2 monochrome digital camera; 1–3 min were needed to generate a grid-map for Au-HZBII or Au-EM finder grids, respectively.

After in vivo imaging (<1 min), culture grids were fixed by plunge-freezing with an EM CPC vitrification unit (Leica Microsystems). Vitrified grids were transferred in liquid nitrogen to the cryo-correlative cooling stage (Linkam Scientific Instruments) to hold samples at a stable −190 °C during analysis. The cryo-stage was inserted into an AxioScope A1 (Carl Zeiss) epifluorescence microscope with a N-Achroplan 10×/0.25 Ph1 objective and imaged with a CCD AxioCam ICm1 (Carl Zeiss).

Cryo-fluorescence correlative microscopy was used to pre-select vitrified samples and map the position of cells containing nanoparticles. Selected samples were then transferred to the synchrotrons at liquid nitrogen temperature.

### Soft X-ray cryo-tomography

Holey carbon-coated (R 2/2; Quantifoil) Au-EM finder grids or Au-HZBII special grids for ALBA and HZB-BESSYII, respectively, were pre-visualised on-line with a visible light microscope integrated within the X-ray microscope to correlate cell position with epifluorescence and cryo-epifluorescence images. At the Mistral beamline, zero degree soft X-ray projection mosaics were acquired to evaluate sample conditions (vitrification and thickness). Tilt series were acquired at 520 eV photon energy from −70° to 70° at 1° interval, using a 40-nm zone plate. Exposure time depends on sample thickness, and ranged from 1 to 4 s. Final image pixel size was 15.56 and 11.5 nm for HZB-BESSYII and ALBA microscopes, respectively. At HZB-BESSYII, we acquired 24 datasets at 24 h. At ALBA, we acquired 69 tilt series as follows: 21 at 3 h, 13 at 6 h, 20 at 12 h, 7 at 24 h and 8 from control cells (no SPION incubation).

Tilt series were normalised to the flatfield using the XMIPP 3 software package [26], aligned with IMOD [27] and reconstructed with the TOMO3D software, 30 iterations simultaneous iterative reconstructive technique (SIRT) algorithm [28]. Semiautomatic segmentation of volumes was carried out with Amira (FEI), and volumes were represented with Chimera [29].

### Statistical analysis

Five cells per treatment were used to extract 3D data. The coordinates for SCVs, extracted manually, and their distances to the nuclear membrane were collected using IMOD [30]. SCV morphology was simplified to spheres to measure vesicle diameters, which include membrane contour when detectable, as well as high-absorption voxels. The data were analysed statistically using the “R” program [31].

## Additional files

10.1186/s12951-016-0170-4 Uptake dynamics of SPION-incubated MCF-7 cells analysed by confocal time-lapse video microscopy. Images were taken every 2.5 min during a 3-h incubation with SPION (fluorescence and reflected light).
10.1186/s12951-016-0170-4 Correlative microscopy in MCF-7 cells incubated with SPION for 24 h. Correlation between a confocal plane of a MCF-7 cell incubated with SPION for 24 h (A) stained with DAPI (blue), LysoTracker Red (red) and backscattered light (B). Cells were fixed in 2 % glutaraldehyde (10 min). Overlay of A and B is represented in C. Bar for A–C, 10 μm. Correlation of cryo-epifluerescence LysoTracker Red signal of an MCF-7 cell incubated with SPION for 24 h (D) and a projection of a cryo-SXT reconstructed 3D volume of the same cell (E). Overlay of D and E is shown in F. Bar for D–F, 5 μm. Correlation between epifluorescent signal of a live MCF-7 cell sample incubated with SPION for 24 h (G) stained with DAPI (blue), LysoTracker Red (red) and TEM micrograph of a thin section of the same sample vitrified and cryo-substituted (H) as described [14]. Overlay of G and H is shown in I. Bar in G–I, 5 μm.
10.1186/s12951-016-0170-4 Comparison of epifluorescence images of cells before and after vitrification. A) Epifluorescence image of live MCF-7 cell incubated with SPION (24 h) and stained with LysoTracker Red. B) Cryo-epifluorescence image of the same area in A after vitrification. Insets show higher magnification details. Bar in A, B, 50 μm. Bar in insets, 15 μm.
10.1186/s12951-016-0170-4 Cryo-SXT correlative workflow at the MISTRAL beamline at ALBA. Images correspond to an MCF-7 control cell and cells incubated with SPION (0.25 mg ml^−1^) for 3, 6, 12 and 24 h. Left, cryo-epifluorescence micrograph of areas with LysoTracker Red- and DAPI-labelled cells. Bar, 20 μm. Centre, cryo-soft X-ray projection mosaic of the area in the yellow squares in the left column. Bar, 5 μm. Right, central sections of reconstructed tomograms of the area in yellow squares in the central column. Bar, 2 μm.
10.1186/s12951-016-0170-4 Volumetric representation of the cryo-SXT tomogram in Fig. 2d. High-absorption vesicles (red), segmented applying a threshold adapted to the volume containing the highest densities, are condensed near the nucleus (blue), displacing the mitochondrial network (yellow). Grey, filaments; green, plasma membrane. Field of view, 16.5 μm × 15.6 μm.
10.1186/s12951-016-0170-4 Histograms showing distributions of SPION-containing vesicle size, distance to the nucleus, and distance to neighbouring endosomes at different incubation times. Y axis, frequency of events. A) Diameter. All distributions differ, according to Kolmogorov–Smirnov test. B) Distance to the nucleus. C) Distance to the four nearest SCV.

## Abbreviations

3D: three-dimensional
Cryo-SXT: cryo-soft X-ray tomography
DIC: differential interference contrast
SPION: superparamagnetic iron oxide nanoparticles
MRI: magnetic resonance imaging
CLXST: correlative light-soft X-ray tomography
CLEM: correlative light-electron microscopy
SCV: SPION-containing vesicle
PES: polyethersulphone
SIRT: simultaneous iterative reconstructive technique
TEM: transmission electron microscopy
