# Supplementary figures and images for: Cryo-soft X-ray tomography as a quantitative three-dimensional tool to model nanoparticle:cell interaction
Source: J Nanobiotechnology. 2016 Mar 3;14:15. doi: 10.1186/s12951-016-0170-4 (PMC4778319; doi:10.1186/s12951-016-0170-4)

CONFOCAL

CLSXT

CLEM

Fluorescence

**A****D****G**

SPION Signal

**B****E****H**

Overlay

**C****F****I**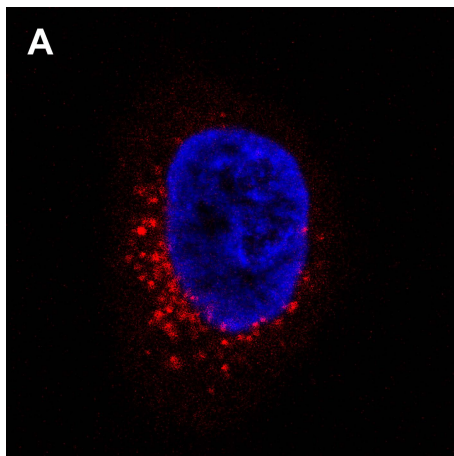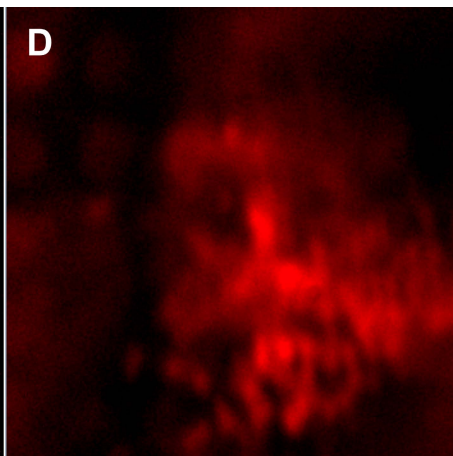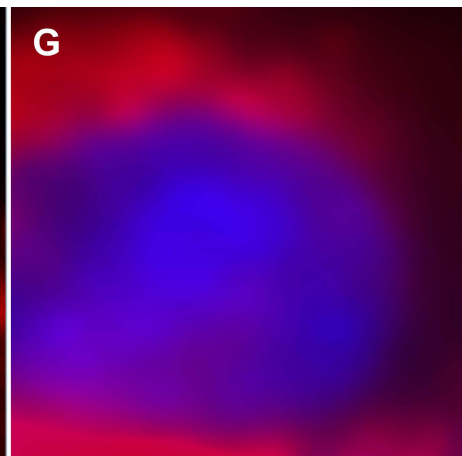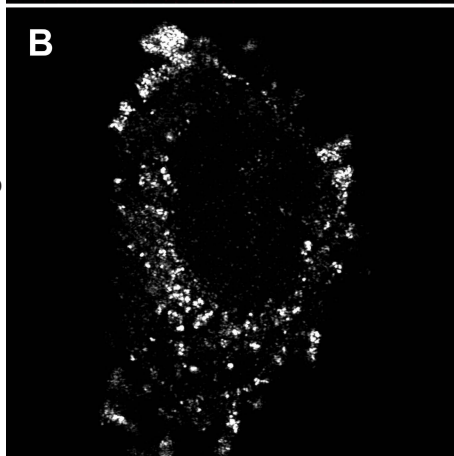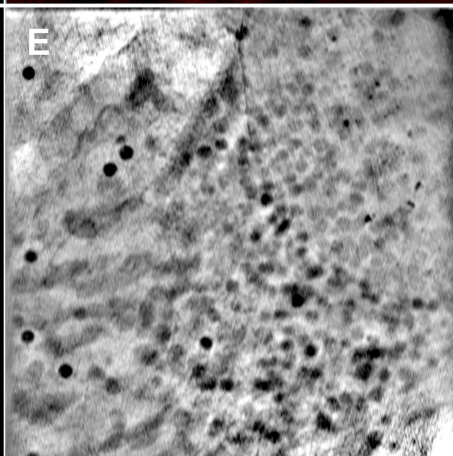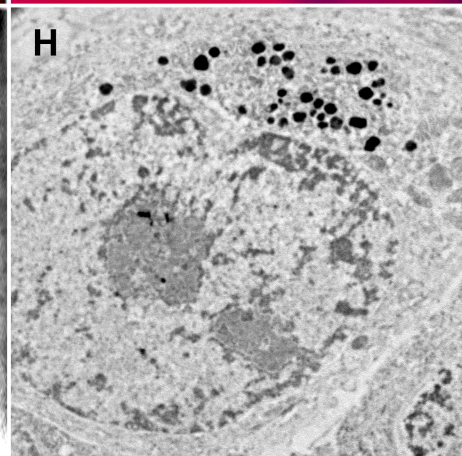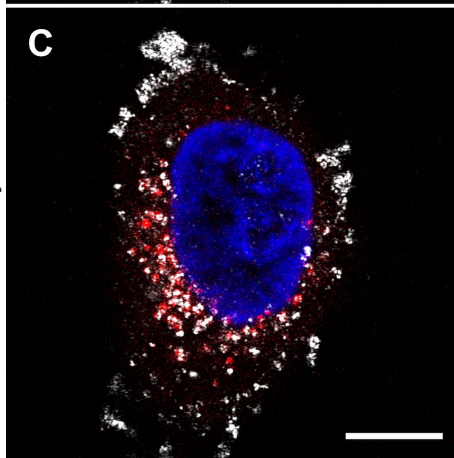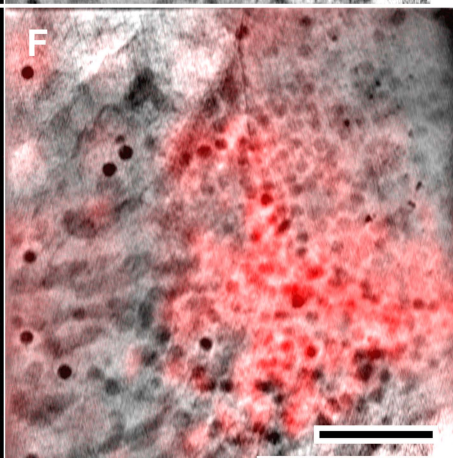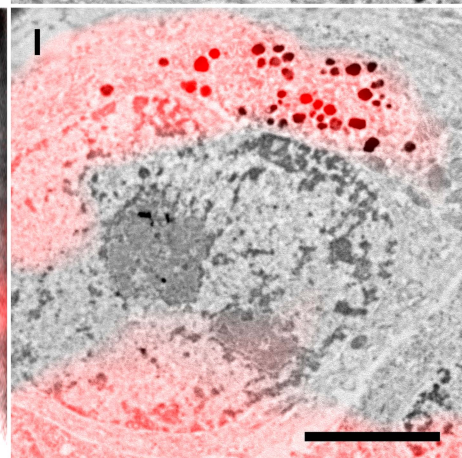

Supplement: Supplementary file 2 — 10.1186/s12951-016-0170-4 Correlative microscopy in MCF-7 cells incubated with SPION for 24 h. Correlation between a confocal plane of a MCF-7 cell incubated with SPION for 24 h (A) stained with DAPI (blue), LysoTracker Red (red) and backscattered light (B). Cells were fixed in 2 % glutaraldehyde (10 min). Overlay of A and B is represented in C. Bar for A–C, 10 μm. Correlation of cryo-epifluerescence LysoTracker Red signal of an MCF-7 cell incubated with SPION for 24 h (D) and a projection of a cryo-SXT reconstructed 3D volume of the same cell (E). Overlay of D and E is shown in F. Bar for D–F, 5 μm. Correlation between epifluorescent signal of a live MCF-7 cell sample incubated with SPION for 24 h (G) stained with DAPI (blue), LysoTracker Red (red) and TEM micrograph of a thin section of the same sample vitrified and cryo-substituted (H) as described [14]. Overlay of G and H is shown in I. Bar in G–I, 5 μm. [file 12951_2016_170_MOESM2_ESM.pdf]

*in vivo* fluorescent signal

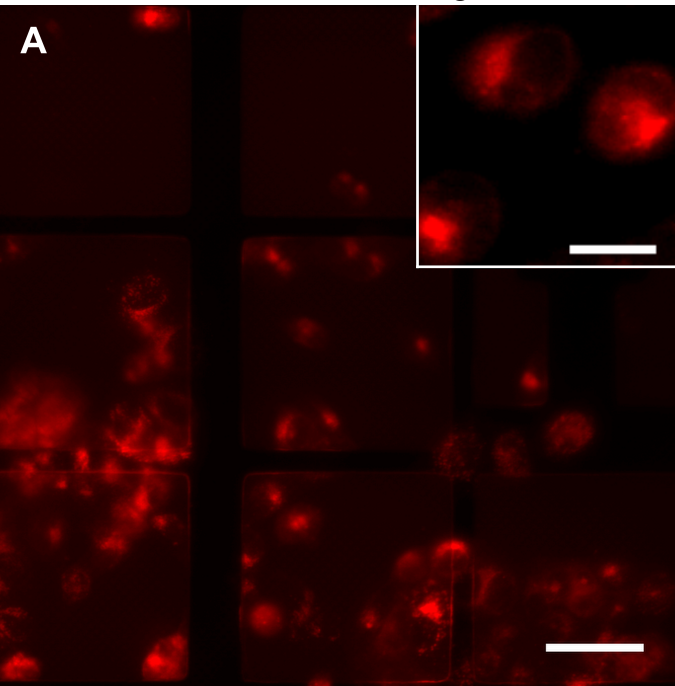

cryo-epifluorescent signal

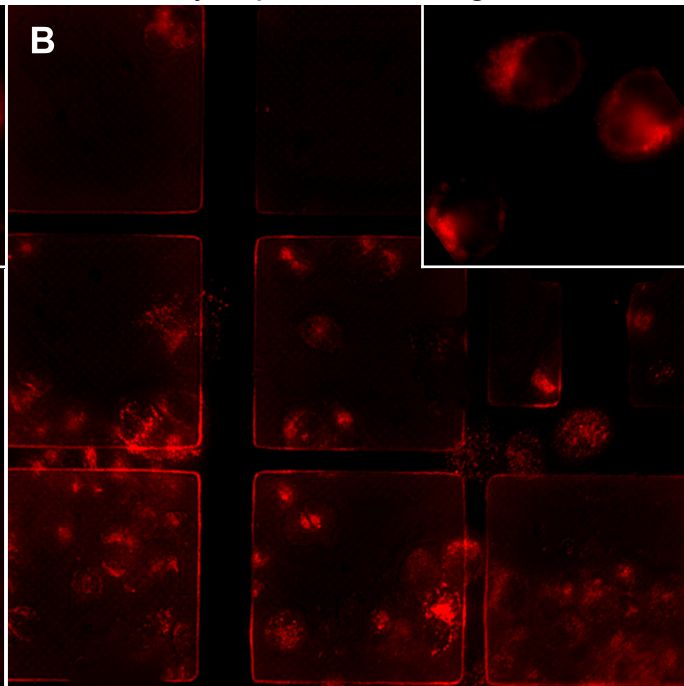

Supplement: Supplementary file 3 — 10.1186/s12951-016-0170-4 Comparison of epifluorescence images of cells before and after vitrification. A) Epifluorescence image of live MCF-7 cell incubated with SPION (24 h) and stained with LysoTracker Red. B) Cryo-epifluorescence image of the same area in A after vitrification. Insets show higher magnification details. Bar in A, B, 50 μm. Bar in insets, 15 μm. [file 12951_2016_170_MOESM3_ESM.pdf]

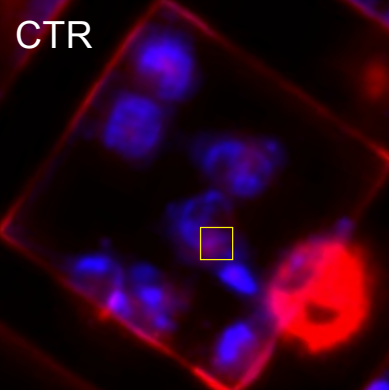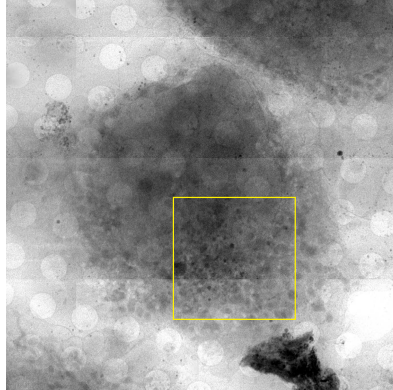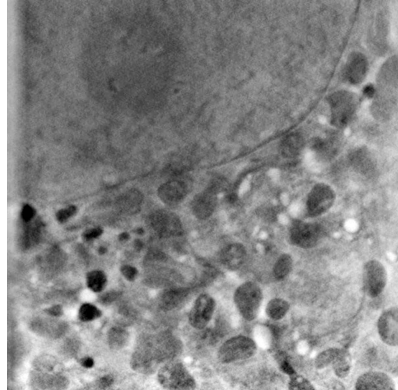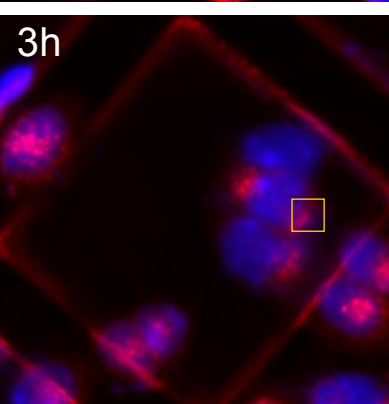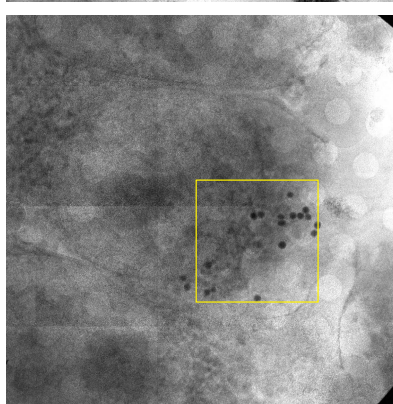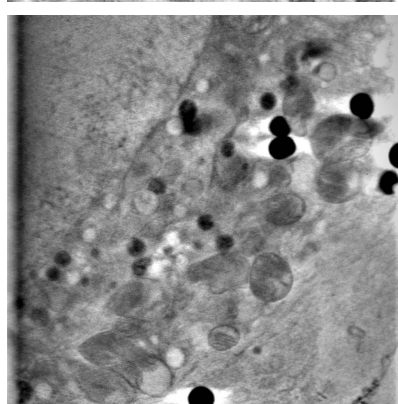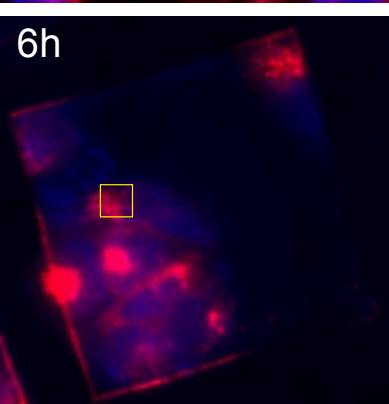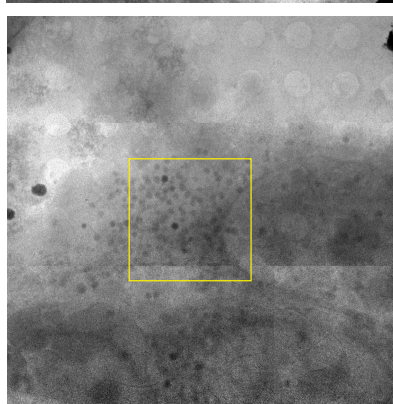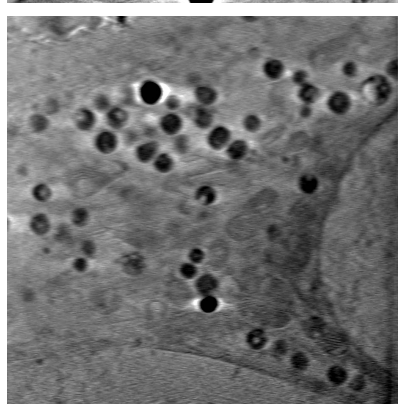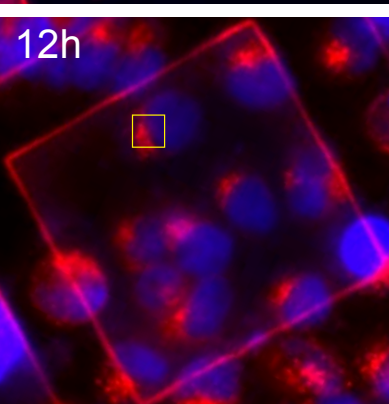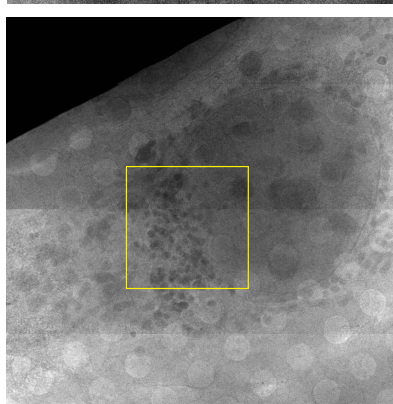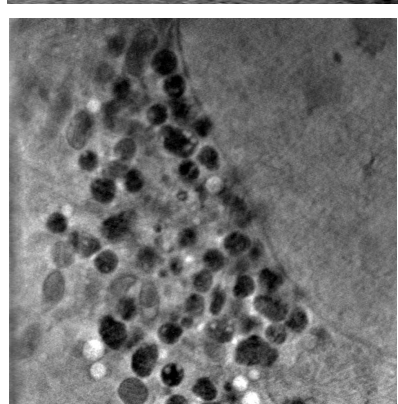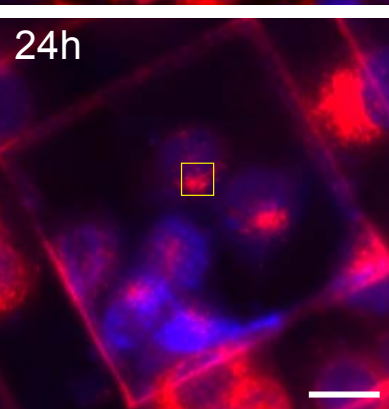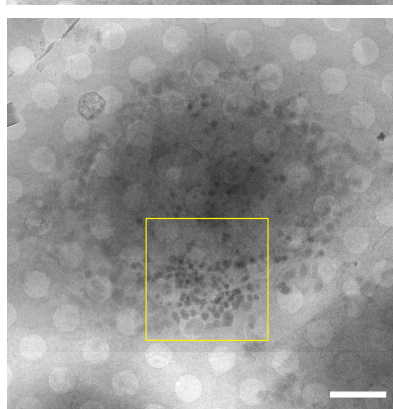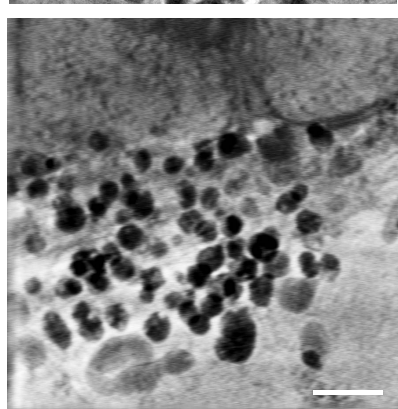

Supplement: Supplementary file 4 — 10.1186/s12951-016-0170-4 Cryo-SXT correlative workflow at the MISTRAL beamline at ALBA. Images correspond to an MCF-7 control cell and cells incubated with SPION (0.25 mg ml−1) for 3, 6, 12 and 24 h. Left, cryo-epifluorescence micrograph of areas with LysoTracker Red- and DAPI-labelled cells. Bar, 20 μm. Centre, cryo-soft X-ray projection mosaic of the area in the yellow squares in the left column. Bar, 5 μm. Right, central sections of reconstructed tomograms of the area in yellow squares in the central column. Bar, 2 μm. [file 12951_2016_170_MOESM4_ESM.pdf]

A

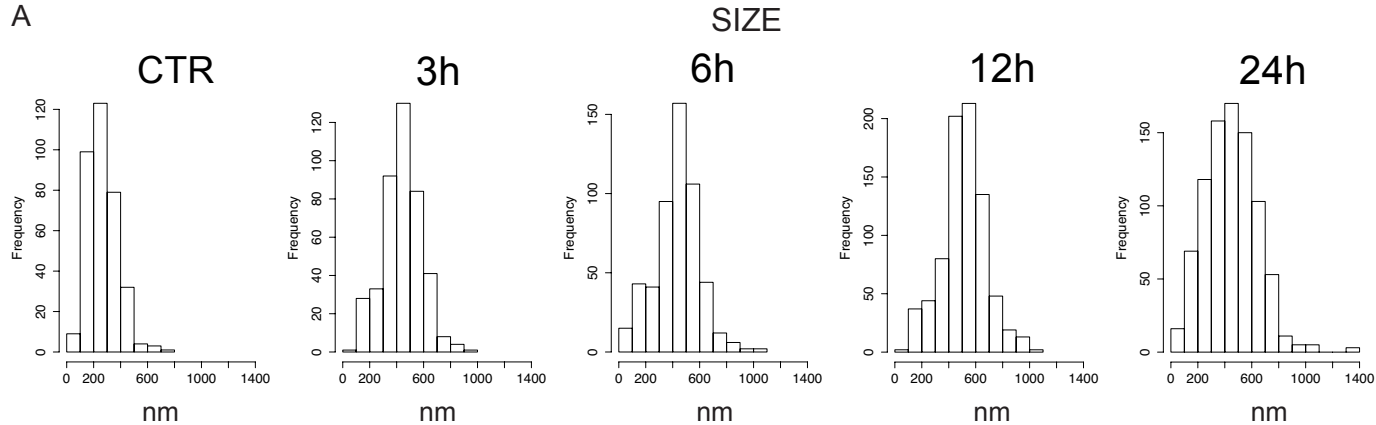

B

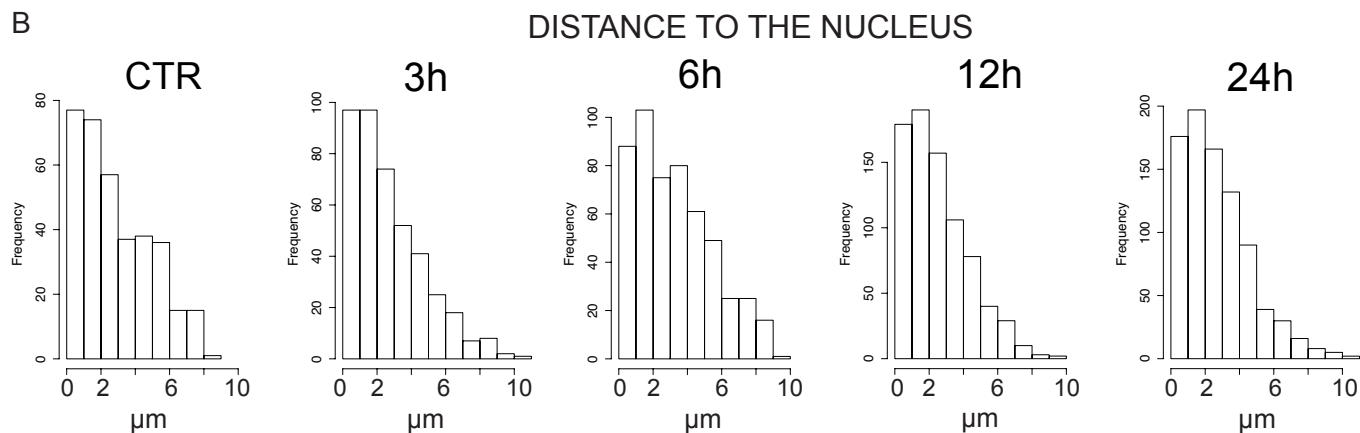

C

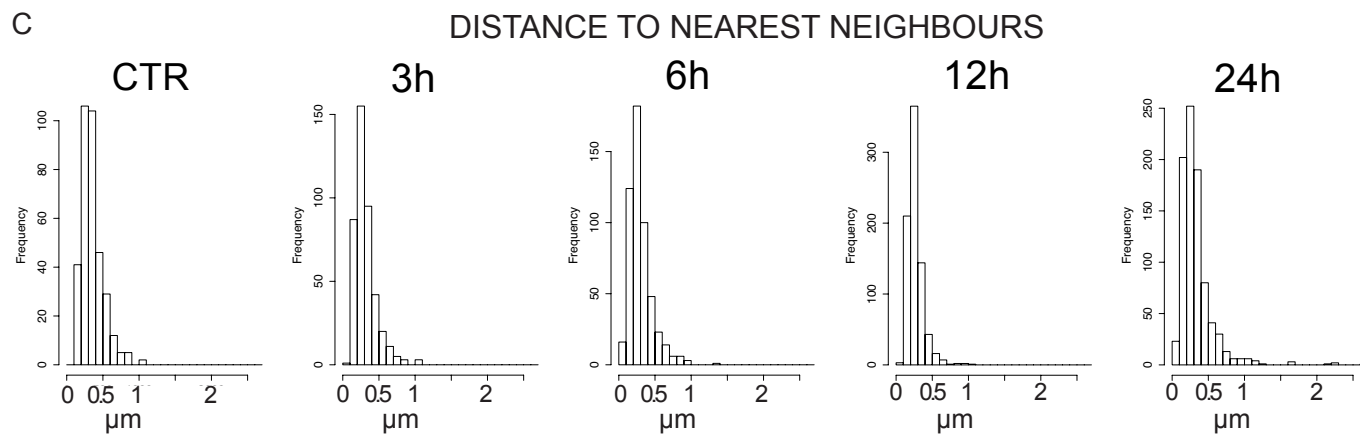

Supplement: Supplementary file 6 — 10.1186/s12951-016-0170-4 Histograms showing distributions of SPION-containing vesicle size, distance to the nucleus, and distance to neighbouring endosomes at different incubation times. Y axis, frequency of events. A) Diameter. All distributions differ, according to Kolmogorov–Smirnov test. B) Distance to the nucleus. C) Distance to the four nearest SCV. [file 12951_2016_170_MOESM6_ESM.pdf]
